# Retraction: Armed violent conflict and healthcare-seeking behavior for maternal and child health in sub-Saharan Africa: A systematic review

**DOI:** 10.1371/journal.pone.0362033

**Published:** 2026-11-04

**Authors:** The PLOS One Editors

Following the publication of this article [1], concerns were raised about the methodology. Specifically:

- Supporting information file S1 Checklist is the PRISMA-P checklist for systematic review protocols, not the standard PRISMA checklist for the systematic review itself. Furthermore, the checklist was not filled out with page numbers or section titles where the information can be found in the manuscript or article.
- Articles published in 2023 were excluded without adequate justification.
- The stated aims, objectives, and research question are inconsistent as to whether the review is focused on describing the body of literature and identifying knowledge gaps, or assessing the impacts of exposure to armed violence conflicts on health seeking behavior in sub-Saharan Africa.
- The methods of data synthesis are not adequately described. Furthermore, the article lacks systematic synthesis of the evidence. Instead of critically analyzing and synthesizing findings across outcomes, the authors presented a directional count of study outcomes accompanied by selective (non-comprehensive), study-by-study (as opposed to per outcome) narrative descriptions, without comparing contextual factors or study quality to explain why results varied.
- The authors treated publication bias as an individual study risk-of-bias domain rather than an outcome-wide certainty domain under the quality or certainty of evidence framework (GRADE) (Fig 3 in [1]). GRADE assessments were applied to individual studies as well, rather than evaluating per outcome across studies (Fig 3 in [1]). Furthermore, GRADE certainty ratings were incorrectly applied, assigning a “high” quality rating to nearly all observational and qualitative studies without documenting any justification for upgrading, which gives a false impression of high evidence certainty.

The corresponding author provided a completed PRISMA 2020 checklist with page numbers, and stated that the review “sought to answer the question around what evidence has been reported regarding the relationship between armed conflict exposure and maternal and child health-care seeking behaviors in sub-Saharan Africa”. However, this is not consistent with the research question and PICO framework posed in the Data sources and search strategy subsection of the Methods section. The corresponding author also stated that “data were synthesized narratively to identify recurring themes”, and that “the evidence synthesis is presented in the Results section”. However, the *PLOS One* Editors remain concerned that the methods of synthesis are not sufficiently well-described to be reproducible, and conclusions relating to the impact of conflict on healthcare seeking behavior, comparisons between the impact on maternal vs. child health, and explanatory factors are not adequately supported by the Results.

In light of the above unresolved concerns that question the validity of the published results and conclusions in [1], the *PLOS One* Editors retract this article.All authors did not agree with the retraction.
